# Circulative Transmission of Cileviruses in *Brevipalpus* Mites May Involve the Paracellular Movement of Virions

**DOI:** 10.3389/fmicb.2022.836743

**Published:** 2022-04-06

**Authors:** Aline Daniele Tassi, Pedro Luis Ramos-González, Thais Elise Sinico, Elliot Watanabe Kitajima, Juliana Freitas-Astúa

**Affiliations:** ^1^Laboratório de Biologia Molecular Aplicada, Instituto Biológico, São Paulo, Brazil; ^2^Escola Superior de Agricultura Luiz de Queiroz (ESALQ), Universidade de São Paulo, Piracicaba, Brazil; ^3^Centro de Citricultura Sylvio Moreira, Cordeirópolis, Brazil; ^4^Embrapa Mandioca e Fruticultura, Cruz das Almas, Brazil

**Keywords:** virus vector relationship, *Kitaviridae*, virus movement, septate junctions, flat mite, citrus leprosis virus C

## Abstract

Plant viruses transmitted by mites of the genus *Brevipalpus* are members of the genera *Cilevirus*, family *Kitaviridae*, or *Dichorhavirus*, family *Rhabdoviridae*. They produce non-systemic infections that typically display necrotic and/or chlorotic lesions around the inoculation loci. The cilevirus citrus leprosis virus C (CiLV-C) causes citrus leprosis, rated as one of the most destructive diseases affecting this crop in the Americas. CiLV-C is vectored in a persistent manner by the flat mite *Brevipalpus yothersi*. Upon the ingestion of viral particles with the content of the infected plant cell, virions must pass through the midgut epithelium and the anterior podocephalic gland of the mites. Following the duct from this gland, virions reach the salivary canal before their inoculation into a new plant cell through the stylet canal. It is still unclear whether CiLV-C multiplies in mite cells and what mechanisms contribute to its movement through mite tissues. In this study, based on direct observation of histological sections from viruliferous mites using the transmission electron microscope, we posit the hypothesis of the paracellular movement of CiLV-C in mites which may involve the manipulation of septate junctions. We detail the presence of viral particles aligned in the intercellular spaces between cells and the gastrovascular system of *Brevipalpus* mites. Accordingly, we propose putative genes that could control either active or passive paracellular circulation of viral particles inside the mites.

## Introduction

Plant diseases caused by *Brevipalpus*-transmitted viruses (BTV) result in non-systemic infections that produce local necrotic and chlorotic lesions on leaves, stems, and fruits (Kitajima et al., 2003, 2010). Early studies based on ultrastructural analyses of BTV-infected tissues revealed two types of viruses which were further recognized as BTV-Cytoplasmic and -Nuclear types (Kitajima et al., 2003). BTV-C and -N have contrasting molecular biology but they still display some common features suggesting a possible convergent evolution (Freitas-Astúa et al., 2018).

**FIGURE 1:**
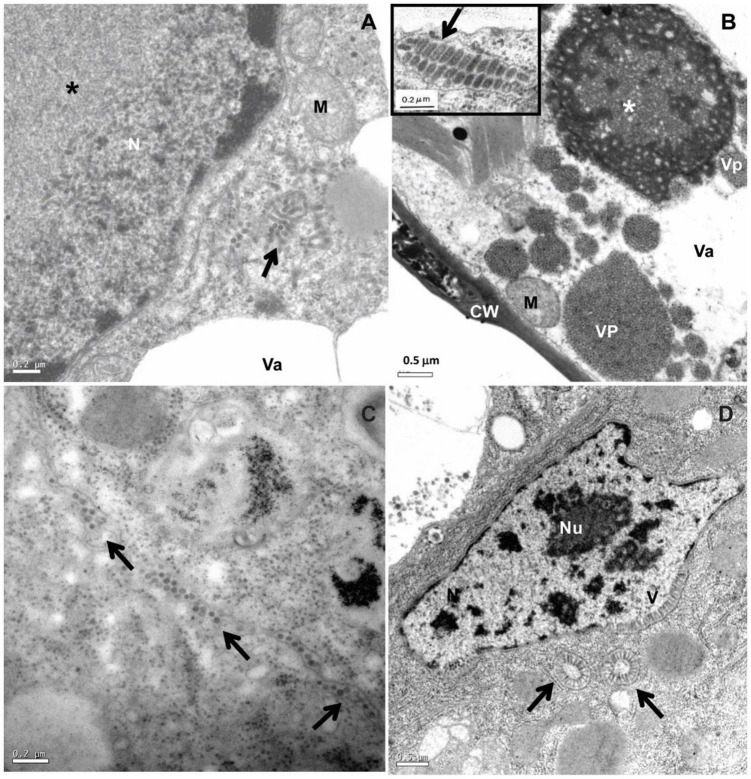
Transmission electron micrographs of BTV-infected leaf lesion cell and mite vector *Brevipalpus yothersi* tissues. **(A)** *Clerodendrum thomsonae* infected by the dichorhavirus clerodendrum chlorotic spot virus (ClCSV), with electron lucent viroplasm (*) and short, rod-like particles (arrow) in the cytoplasm. **(B)** *Ligustrum sinense* infected with the cilevirus passion fruit green spot virus (PfGSV) that induces large electron dense and vacuolated viroplasm (*) in the cytoplasm; short bacilliform virions (VP) are present within endoplasmic reticulum cisternae (insert). **(C)** Sections from adults *Brevipalpus yothersi* collected from *L. sinense* infected with PfGSV showing virus particles (arrows) in the extracellular space. **(D)** Sections from adults *Brevipalpus yothersi* collected from *C. thomsonae*, infected by ClCSV showing evidences of viral replication in their tissues, large nuclei of the anterior podocephalic gland cell, with virus-like particles (V) at the nuclear periphery. These particles are also present in the cytoplasm, forming the spoke wheel configuration (arrows). C, chloroplast; CW, cell wall; M, mitochondrion; N, nucleus; Va, vacuole; Nu, nucleolus.

The infection by BTV-N induces the formation of electron-lucent viroplasms in the nucleus. Virions are naked, short rod-like particles (40 nm × 100-110 nm) that accumulate both in the nucleus and the cytoplasm of plant and mite cells (Freitas-Astúa et al., 2018; Figure 1A). The genomes of BTV-N comprise two ssRNA molecules (∼ 6 kb each) of negative sense with six open reading frames (Dietzgen et al., 2018). Five BTV-N have been molecularly characterized and are definitive members of the genus *Dichorhavirus*, family *Rhabdoviridae* (Dietzgen et al., 2018; Amarasinghe et al., 2019; Kuhn et al., 2020).

Virions of the BTV-C type are short, enveloped bacilliform particles of 70-80 nm wide and 100-120 nm long. Virus particles are commonly found inside cisternae of the endoplasmic reticulum, and form electron-dense, vacuolated inclusions (viroplasms) in the infected plant cell cytoplasm (Figure 1B). BTV-C genomes consist of two (+) sense single-stranded (ss) RNA molecules of ∼5 and 9 kb. They are assigned to the genus *Cilevirus*, family *Kitaviridae* (Freitas-Astúa et al., 2018; Quito-Avila et al., 2021). Citrus leprosis virus C (CiLV-C) is the best-characterized cilevirus at both molecular and epidemiological levels (Chabi-Jesus et al., 2021).

Besides the genus *Cilevirus*, the family *Kitaviridae* comprises other two plant-infecting virus genera: *Higrevirus* and *Blunervirus* (Quito-Avila et al., 2021). Overall, kitaviruses show a heterogeneous genome organization and share a phylogenetic relationship with arthropod-infecting viruses of the groups negevirus (including nelorpiviruses and sandewaviruses), centivirus, and aphiglyvirus (Kondo et al., 2020; Ramos-González et al., 2020).

Few species of *Brevipalpus* are known as vectors of cileviruses and dichorhaviruses (de Lillo et al., 2021). Dichorhaviruses are transmitted in a circulative propagative manner, whereas cileviruses are transmitted in a circulative manner, but whether cileviruses replicate in the mites is still unclear (Roy et al., 2015; Tassi et al., 2017). Little is known about the mode of transmission of negeviruses and kitaviruses other than cileviruses. The isolation of Okushiri virus from mosquito larvae suggests the vertical transmission of negeviruses (Vasilakis et al., 2013; Carapeta et al., 2015; O’Brien et al., 2017). The expansion of perinuclear spaces filled with vesicles or microtubules, sometimes in paracrystalline arrays, and the accumulation of cytoplasmic vacuoles similar to those detected during the alphavirus infection, are indicators of the multiplication of the sandewavirus Tanay virus in C6/36 mosquito cells (Vasilakis et al., 2013; Zhao et al., 2019). *Brevipalpus* mites have been associated with the transmission of hibiscus green spot virus 2, genus *Higrevirus*, and the cilevirus-like hibiscus yellow blotch virus (Melzer et al., 2013; Olmedo-Velarde et al., 2021).

### *Brevipalpus* Mites as Viral Vectors

The genus *Brevipalpus* groups almost 300 valid species (Castro et al., 2020) of obligatory phytophagous, mostly polyphagous red-brownish mites, which are distributed across the subtropical and equatorial regions of the world. *Brevipalpus* mites are flattened, of approximately 0.3 mm long, move slowly, and display five developmental phases, *i*.*e*., egg, larvae, protonymph, deutonymph, and adult (Welbourn et al., 2003; Alberti and Kitajima, 2014; Tassi et al., 2017; Dietzgen et al., 2018).

Data on *Brevipalpus*-dichorhavirus relationships are almost limited to studies derived from the pathosystem orchid fleck virus (OFV)-*B. californicus* (Kondo et al., 2003). Upon acquisition, OFV transmission has a latent period of three weeks, the inoculation access period is approximately 30 min, and viral retention in mites occurs for at least three weeks (Kondo et al., 2003). Nymphs and adults, but not the larvae, have vector activity, suggesting a persistent propagative type of transmission (Kondo et al., 2003).

Different species of *Brevipalpus* colonizing dichorhavirus-infected plants exhibit electron-lucent viroplasms in the nucleus, and short rod-like particles in both the nucleus and the cytoplasm of midgut and anterior podocephalic gland cells (Alberti and Kitajima, 2014; Ramos-González et al., 2018). In the nucleus, viral particles may appear dispersed within nucleoplasm or viroplasm and arranged perpendicularly onto the inner membrane of the nuclear envelope (Figure 1). In the cytoplasm, they are commonly seen associated with endoplasmic reticulum membranes, occasionally radially arranged, forming the so-called “spoke wheel” configuration (Figure 1). The accumulation patterns of viral particles in viruliferous mites are essentially similar to those observed in dichorhavirus-infected plant cells, suggesting that dichorhaviruses replicate in the mite (Kitajima et al., 2003). No accumulation of particles is observed between adjacent cells of dichorhavirus-infected mites.

**FIGURE 2:**
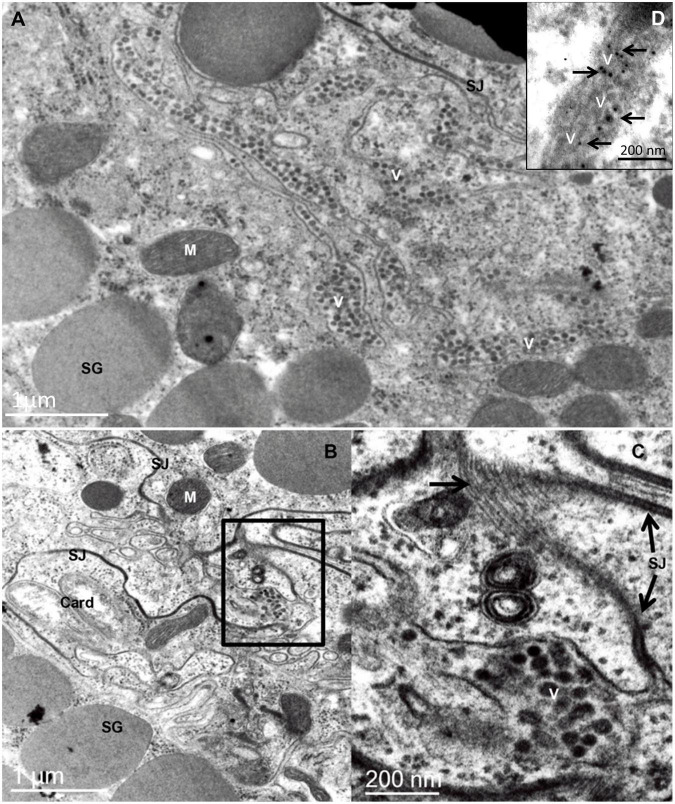
Transmission electron micrographs of sections of the prosomal region of an adult female *Brevipalpus yothersi*, viruliferous for citrus leprosis virus C (CiLV-C). **(A)** Basal part of midgut caeca, showing several rows of virions (V), aligned in the extracellular space formed by four layers of cells. It is presumed that once internalized, crossing the midgut epithelial cell barrier, these particles move passively in the direction of the anterior podocephalic gland (= salivary gland) following the celomic flux, where they will reach the stylet channel, after overtaking the gland cell barrier. **(B)** An area of the branched ceaca, revealing a labyrinth of membranes running between adjacent cells. A small group of virions (V) is present in one of these intercellular spaces. **(C)** An enlarged region of figure B in which septate junctions (SJ) are well depicted. The large arrow points to a tangential section through a septate junction, revealing the rows of intermembrane proteins. **(D)** *in situ* immunolabeling using anti-p29 polyclonal antibody in aldehyde-fixed and LRWhite embedded *B. yothersi* viruliferous for CiLV-C. Card, bacterial endosymbiont Cardinium; M, mitochondrion; SG, secretion granules.

All the active life stages of *B. yothersi* can transmit the cilevirus CiLV-C, but no transovarial passage occurs (Chiavegato, 1996; Tassi et al., 2017). Using common bean (*Phaseolus vulgaris*) as indicator plant (Garita et al., 2013), CiLV-C acquisition and inoculation access periods by *B. yothersi* are 4- and 2 h, respectively, with a latent period of 7 h (Tassi et al., 2017). Once the virus is acquired, *B. yothersi* remains viruliferous for at least 20 days (Tassi et al., unpublished). Viral particles are consistently found between adjacent cells at the basal part of the caeca and in the anterior podocephalic gland of mites (Figure 1C; Alberti and Kitajima, 2014). The load of viral particles in the intercellular spaces seems to increase proportionally with the number of acquisitions (Tassi et al., 2017). Frequently, lines of viral particles are interrupted by septate junctions (Figures 2B,C). Differently from what is easily seen in plant cells infected by CiLV-C, viroplasms are not observed in CiLV-C viruliferous mites. To improve the viral identification, *in situ* immunogold labeling using polyclonal antibodies against the P29 protein of CiLV-C (Calegario et al., 2013) was carried out as previously described for plant tissues (Calegario et al., 2013) and *Brevipalpus* mites (Alberti and Kitajima, 2014). Sets of virion particles aligned up to 10 μm in length were detected in paracellular spaces of *Brevipalpus* mites that fed on CiLV-C-infected oranges (Figure 2).

The detection of anti-genomic (complementary strand) RNA of the cileviruses CiLV-C and CiLV-C2 in viruliferous *B. yothersi* was considered evidence of cilevirus replication within the vector (Roy et al., 2015). However, anti-genomic RNA molecules of CiLV-C may have arisen in the mite body upon feeding on infected plants or may have been generated as a result of self-primed genomic molecules during the RT-PCR detection. Therefore, further assays, including new controls, a time-course experiment, and the search for putative replication sites in specific mite tissues not yet visualized by transmission electron microscopy are ongoing (Tassi et al., unpublished).

## Cileviruses Movement Within Their Mite Vectors: A Critical Evaluation of the Alternatives

### Transcytosis in Circulative Non-propagative Viruses

In addition to whether cileviruses replicate in their mite vectors or not, the mechanisms that promote virion internalization, movement, and their release into the stylet canal also remain uncertain. Transcytosis is a cellular mechanism in which extracellular materials, enclosed in vesicles generated by endocytosis, move across the cell and eject the content in the distal section of the cells by exocytosis (Whitfield et al., 2015). Transcytosis has been also described as a form of circulation of plant viruses in their vectors, but the underlying mechanisms are not fully elucidated (Brault et al., 2007; Hogenhout et al., 2008; Blanc et al., 2014; Di Mattia et al., 2020). The internalization in the vector body of several viruses of the families *Luteoviridae, Geminiviridae*, and *Nanoviridae* occurs without replication of the viral genome. Virus particles are transported across cells into membrane vesicles, preventing any contact between viruses and the insect cell cytoplasm in the epithelia of the gut and salivary gland (Brault et al., 2007; Hogenhout et al., 2008; Blanc et al., 2014). The vesicles formed during transcytosis seemingly follow the early endosomal pathway before the appearance of non-coated tubular vesicles. Inside these vesicles, virions likely reach the basal membrane and exit the gut cells into the hemolymph (Ali et al., 2018). A transcytosis process is also observed when luteovirids cross the cellular barrier of the accessory salivary gland (Brault et al., 2007). For cileviruses, however, although transcytosis should not be completely disregarded, it seems an unlikely route of circulation in *Brevipalpus* mites. Viral particles have been observed neither inside cells of the anterior midgut epithelium nor in cells of anterior and dorsal podocephalic glands of mites feeding on cilevirus-infected plants (Alberti and Kitajima, 2014).

It is important to notice that anatomic differences between *Brevipalpus* mites and insects may account for the nonexistence of transcytosis in these mites. During feeding, *Brevipalpus* mites use stylets to perforate the epidermal layer of plant organs and reach the underlying parenchymal cell content after punctuating its wall and membrane. Saliva produced by the anterior podocephalic gland is injected to pre-digest the cellular content (Alberti and Kitajima, 2014). The ingested material then follows to the esophagus that crosses the synganglion and ends into the anterior midgut through a small valve, the ventriculus, which consists of a small lumen and the highly branched caeca (Alberti and Kitajima, 2014). The caeca, formed by large epithelial cells, extend both to anterior and posterior parts of the mite body, occupying every space among the organs, producing a complex labyrinth of cell membranes and intercellular spaces, many of which are joined by septate junctions (Figures 2A-C), leaving the hemolymph confined to small and restricted cavities. This complex of cells comprises the so-called gastrovascular system which may directly irrigate several organs with digested nutrients and, probably, virus particles in viruliferous mites (Alberti and Kitajima, 2014). *Brevipalpus* mites lack a pulsating organ, so the circulation of the hemolymph depends on the movement generated by their muscles and internal organs, diverging from insects that have a circulatory system and, therefore, a more active circulation of nutrients and other fluids (Alberti and Coons, 1999).

### Paracellular Route of CiLV-C in *Brevipalpus yothersi*: A Hypothesis

The persistent circulatory transmission of cileviruses by *Brevipalpus* mites poses a challenge to explain how the cilevirus movement occurs. A raising question is how virions get access from the midgut lumen to the hemolymph space, and later to the stylet channel, since two epithelial barriers hamper it: the midgut (caeca) and the anterior podocephalic gland. Ultrastructural observations of viruliferous *B. yothersi* mites feeding on CiLV-C-infected plants reveal the presence of viral particles between cells (Alberti and Kitajima, 2014), which led us to ponder the existence of a paracellular pathway of virion movement within its vector.

In the epithelium of invertebrates, occluding structures named septate junctions (SJ) act as a barrier that separates distinct compartments, limiting the paracellular passage of fluids (Izumi and Furuse, 2014). Unlike tight junction (TJ), the structure sealing the apical part of epithelial cells in the vertebrates, the SJ forms circumferential belts around the apicolateral regions of the cells. Visually, they appear as a ladder-like septum, with 15-20 nm of spacing (Izumi and Furuse, 2014). Studies of the morphophysiology of the SJ in the fly *Drosophila melanogaster* exposed two types of SJ, i.e., the pleated septate junctions (pSJ), present in ectodermal-derived epithelium, i.e., epidermis, fore- and hindgut, salivary gland, etc., and the smooth septate junctions (sSJ), which occurs in the endodermal-derived epithelium, i.e., midgut, gastric caeca (Izumi and Furuse, 2014; Hall and Ward, 2016). Mutations of the SJ proteins may be lethal or produce functionally deficient junctions, but the exact mechanisms underlying the transient opening of SJ have not been described. To our knowledge, there are no reports of arthropod viruses interacting with tight or septate junctions.

In vertebrates, TJs could act as physical barriers from the innate immune defense system, especially on the respiratory tract. The coordination of TJ opening is mediated by chemical signals and membrane receptors, as happens during the paracellular passage of lymphocytes through the walls of capillary vessels (Yonekawa and Harlan, 2005; Yumine et al., 2019). In humans, the permeability of TJ between cells of the wall of the digestive tract is mediated by zonulin, a pre-haptoglin protein, and by gliadin, a component of gluten, in patients suffering from celiac disease (Fasano, 2012). Similarly, to replicate or transit through epithelia, viruses take advantage of the structural proteins that form the TJ and adherent junctions (AJ) as their receptors (Mateo et al., 2015). Viruses evolved selecting strategies that could counter the antiviral function of TJs, by degradation processes, e.g., it is suggested that mosquito-borne viruses like west nile virus and Japanese encephalitis virus establish infections in vertebrate hosts by degrading TJ molecules to disrupt the epithelial barriers (Medigeshi et al., 2009; Yumine et al., 2019).

The gastrovascular system of *Brevipalpus* mites, composed of the highly branched caeca that fill all spaces left by other tissues on the hemolymph, may serve as a pathway to the circulation of viruses inside the mites.

**FIGURE 3:**
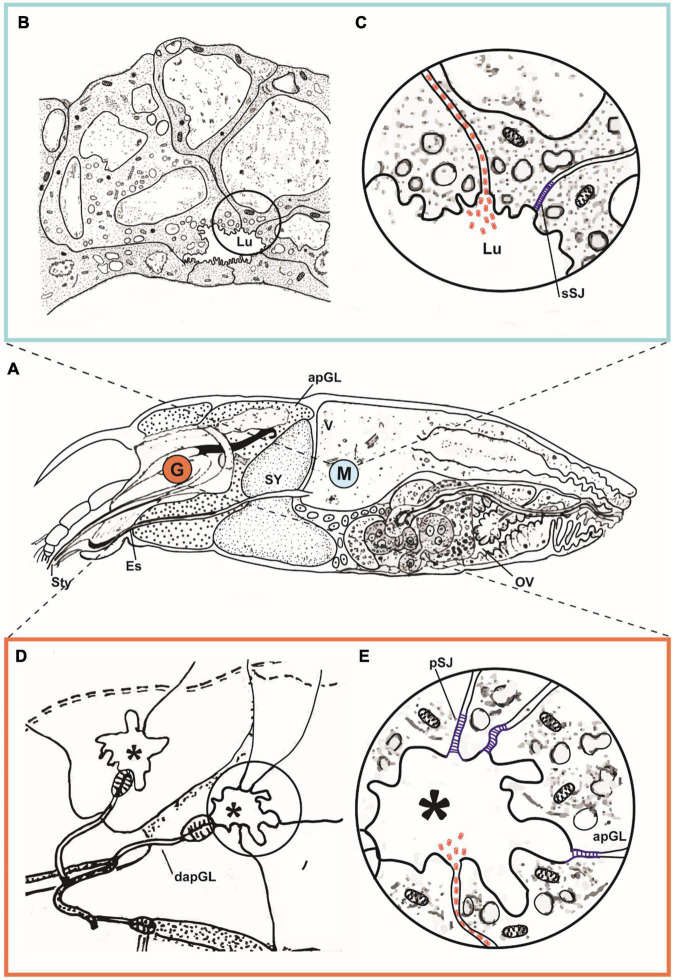
Hypothetical pathway of cileviruses circulating within the mite vector. **(A)** Schematic representation of sagittal section of an adult female of *Brevipalpus yothersi*, revealing details of its internal anatomy [stylet complex, prosomal glands, synganglion, digestive tract (ventriculus, caeca), ovary]. Details of ventriculus (V) and anterior podocephalic gland (apGL) are shown indicated in the marked area M and detailed in **(B,C)**. Portion of the anterior podocephalic gland (equivalent to salivary gland) marked by G, is detailed in **(D,E)**. **(B)** Part of ventriculum (V) exhibiting epithelial cells of caeca with small lumen (Lu). In the detail **(C)**, hypothetical transitional opening of the smooth septate junction (sSJ) at the apical part of the epithelial cell, induced by the presence of viral particles represented in red. Virions gain access to the intercellular space (Lu), being carried out by the celomic flux, to the apical part of the anterior podocephalic gland. **(D)** Schematic drawing of collecting reservoir (*) of the apGL secretions. **(E)** Detail depicted in **(D)**. Glandular cells are held together by pleated septate junctions (pSJ) represented in blue, which hypothetically open due to the presence of viral particles (in red) arriving by the intercellular space, releasing them into the reservoir (*), and subsequently to the stylet channel. dapGL, duct of the anterior podocephalic gland (adapted from Alberti and Kitajima, 2014).

Based on our transmission electron microscopy studies following the methodology proposed by Alberti and Kitajima (2014), observations of the accumulation of virus particles between cells and the possible role of SJ limiting or coordinating the circulation of viruses to new tissues, we pose alternative hypotheses to explain virus-gastrovascular system interaction including either passive or active mechanisms. The passive interaction involves the SJs transient openings which allow the transport of nutrients between cells. Following the flow, the virus could reach the spaces between cells, leading to a circulation of virus particles within the hemolymph. Alternatively, virus-encoded proteins could recognize the SJs components and induce transient openings, allowing the passage of viruses in an active process, as seen in some vertebrates-infecting viruses, or even in other physiological or pathological processes, i.e., zonulin and gliadin-like interaction (Figure 3). The viral protein P61, a putative membrane glycoprotein, or P24, a putative viral structural protein, might trigger a transient local opening of the SJ, ensuing the paracellular traffic of cileviruses in *Brevipalpus* mites.

### Orthologue Proteins of SJ and sSJ Factors in Mites

The involvement of TJs and AJs in cell-to-cell viral movement and their role as receptors have been reported for human viruses (Cifuentes-Munoz et al., 2020), but there is no information on viruses using this route in arthropods. In humans, for instance, after primary infection of the respiratory airway, measles virus, family *Paramyxoviridae*, spreads laterally into the epithelium *via* AJs (Mühlebach et al., 2011; Nakatsu et al., 2013). Besides, human TJ components act as viral receptors, e.g., the glycoprotein of hepatitis C virus (HCV) uses occludin and claudin as co-receptors to enter hepatocytes (Reynolds et al., 2008; Timpe et al., 2008; Brimacombe et al., 2011; Yumine et al., 2019), the coxsackieviruses (positive-strand RNA viruses) and adenoviruses (double-stranded DNA viruses), although exploring different strategies, target the integral chimeric antigen receptor (CAR) protein associated with TJ (Bergelson et al., 1997; Bergelson, 2009; Mateo et al., 2015), reoviruses use the TJ protein junctional adhesion molecule A (JAM-A) as receptor (Barton et al., 2001), claudin 1 is involved on dengue virus entry by the interaction of the viral protein prM and TJ component (Che et al., 2013).

Proteins of the family LY6_uPAR, also called three-finger proteins (TFP), are found associated with the membrane by a glycosylphosphatidylinositol anchor and play essential roles in cell adhesion, signaling, and lipid metabolism (Vasilyeva et al., 2017). TFPs display one or several domains consisting of 60-90 amino acids which have an β-structural core stabilized by a system of four invariant disulfide bonds. Proteins with similar structural characteristics have been found in the mite *Tetranychus urticae* and insects (Grbić et al., 2011; Zhang et al., 2019). In flies, TFP-like proteins are involved in the formation of SJ adhesion structures, suggesting a common ancient role for these proteins in arthropods (Hijazi et al., 2009).

In this study, based on literature review and Blast N search^1^, we were able to identify putative homologs of proteins that regulate SJ and sSJ in *Drosophila* encoded by the genomes of *T. urticae* (the most studied mite in the superfamily Tetranychoidea) and *B. yothersi* (Table 1). One of the genes identified is the polychaetoid (*pyd*) gene, which is a recognized homolog of zonulin (Seppa et al., 2008; Choi et al., 2011; Djiane et al., 2011). The study of the transcriptomic profile of these genes in viruliferous mites will probably add hints on SJ and sSJ opening processes in mites and their possible relationship with cileviruses.

**TABLE 1:** Genes of *Drosophila melanogaster* involved in SJ and sSJ regulation and their respective orthologs in *Tetranychus urticae* and *Brevipalpus yothersi*.

Gene name			Function in *Drosophila*			References
			
*Drosophila*	*E*-value	*T. urticae*	*E*-value	*B. yothersi*		
Anakonda	0.0	tetur04g07130.1	0.00	bryot81g00190	Putative transmembrane scavenger receptor-like protein that is essential for the maturation SJ	Byri et al., 2015; Hildebrandt et al., 2015; Daniel et al., 2018
Gliotactin	2e-147	tetur30g01560.1	0.00	bryot209g00240	Transmembrane protein localized at tricellular junctions that is necessary for septate junction and permeability barrier formation	Auld et al., 1995; Genova and Fehon, 2003; Schulte et al., 2003; Venema et al., 2004
Cora	2e-151	tetur17g00600.1	0.00	bryot168g00070	Required for SJ integrity with a role in cell-cell interactions, vital for embryonic proper development.	Fehon et al., 1994; Lamb et al., 1998; Ward et al., 2001
Mesh	1e-157	tetur08g07660.1	0.00	bryot35g00820	Transmembrane protein component of smooth SJ organization	Izumi et al., 2012; Chen et al., 2018, 2020
Tetraspanin 2A	2e-08	tetur17g03500.1	1.16e-112	bryot101g00020	Component necessary for the assembly of SJ, on the midgut.	Izumi et al., 2016; Xu et al., 2019
Neuroglian (Nrg)	0.0	tetur19g00920.1	0.00	bryot101g00400	Contributes to the formation of SJ in epithelial cells.	Genova and Fehon, 2003; Godenschwege et al., 2006; Williams, 2009
Nervana 2 (nvr2)	3e-80	tetur35g00730.1	9.65e-154	bryot98g00300	Plays an ion-pump-independent role in junction formation and transport on the plasma membrane	Sun and Salvaterra, 1995; Paul et al., 2003
Lethal (2) giant larvae l(2)gl	1e-156	tetur18g00160.1	0.00	bryot15g00230	Regulates cell polarity, asymmetric cell division. Localized in smooth SJ.	Woods and Bryant, 1991
G protein α i subunit (Gαi)	0.0	tetur05g01580.1	0.00	bryot23g00160	Involved in regulating asymmetric cell division. Localized in SJ.	Schwabe et al., 2005
	0.0	tetur04g03270.1	0.00	bryot32g00500		
	0.0	tetur15g03060.1	1.6e-124	bryot13g00350		
Patj	1E-107	tetur27g00480.1	5.3e-94	bryot140g00140	Paly supporting roles in apico-basal cell polarity and stability of adherens junction	Tanentzapf et al., 2000; Nam and Choi, 2006; Sen et al., 2012
P21-activated kinase	1e-12	tetur13g00020.1	3.9e-172	bryot77g00180	Involved in regulation of cytoskeleton, apical junction assembly.	Conder et al., 2004; Koch et al., 2008; Bahri et al., 2010
Ankyrim 2 (Ank2)	0.0	tetur15g02730.1	2.82e-176	bryot07g00160	Cytoskeletal binding protein, plasma membrane-bounded cell projection organization.	Hortsch et al., 2002; Koch et al., 2008; Bulat et al., 2014
Polychaetoid (pyd)	0.0	tetur33g01420.1	0.00	bryot71g00250	Broadly acting protein that is associated with multiple proteins at the surface and within the cytoskeleton	Seppa et al., 2008; Choi et al., 2011; Djiane et al., 2011

## Discussion

Virion particles of CiLV-C are routinely observed between the membrane of adjacent cells of *B. yothersi* (Alberti and Kitajima, 2014). In contrast, neither virions nor structures such as viroplasms, commonly associated with viral multiplication, have been observed inside mite cells. In this context, this study presents elements that guided us to pose the hypothesis of the paracellular movement of CiLV-C inside *Brevipalpus* mites. This unconventional viral movement has been described in the circulation of several viruses in localized organs by inducing disruptions of TJ in vertebrates. An example is the coronavirus SARS-COV-2, which is favored by the disruption of the airway epithelium. This process facilitates the virus paracellular spread into other tissues besides the translocation of endothelial cells (Tugizov, 2021). A similar mechanism is triggered by the rotavirus VP4 capsid protein. VP4 interacts with zonulin 1, occludin, and claudin, stimulating their redistribution and granting access to the junctional areas, which promotes viral spread in a paracellular way (Nava et al., 2004; Tugizov, 2021).

In phytopathosystems comprising (+)ssRNA viruses, a common virus-vector mode of transmission is circulative non-replicative (Hogenhout et al., 2008; Whitfield et al., 2015; Kondo et al., 2019). In many of these systems, the ingested virions must pass through vector cell barriers to reach the salivary glands, including the gut and hemocoel, involving specific interactions between the virus and vector membrane (Bragard et al., 2013; Blanc et al., 2014).

Negeviruses and other insect-borne viruses are likely vertical transmitted to host offprints (Vasilakis and Tesh, 2015; Kondo et al., 2019). Vertical transmission efficiency of plant-infecting (+)ssRNA viruses is generally low due to biological barriers *via* RNA silencing mechanisms that protect plant germ cells against viral infection (Foster et al., 2002; Martín-Hernández and Baulcombe, 2008). As consequence, most of these viruses depend on arthropod vectors for horizontal transmission in a non-replicative manner (Hull, 2013; Whitfield et al., 2015; Andika et al., 2016).

It is speculated that during virus evolution, some viruses, whose ancestors were arthropods-infecting viruses, adapted to plant hosts, but maintained an intimate relationship with those species of arthropods that eventually became their vectors. In these cases, virus circulation and replication are observed within the arthropod and plant hosts, as are the cases of the plant-infecting reoviruses rice dwarf virus and Southern rice black-streaked dwarf virus and their hemipteran vectors *Nephotettix cincticeps* and *Sogatella furcifera*, respectively, or the bunyavirus tomato spotted wilt virus and its thrips vector *Frankliniella dentalis* (Whitfield et al., 2015, 2018; Dolja and Koonin, 2018; Chen et al., 2019; Lefeuvre et al., 2019).

Biological, cytopathic, and molecular data on the cilevirus-mite relationship suggest that these viruses circulate in the vectors, but whether they replicate still needs to be addressed. It has been proposed that the cileviruses have evolved from an arthropod virus ancestor that somehow was able to infect plants after acquiring a movement protein from a plant virus (Ramos-González et al., 2020). The identification of some nege-like viruses infecting plants gives further support to the arthropod-plant host transitional process particularly involving kitaviruses and kita-like viruses infecting arthropods (Morozov et al., 2020). On this basis, it is tempting to speculate that during adaptation to plants, the presumed ancestor of kitaviruses lost arthropod fitness as it gradually adapted to plant hosts, but still, some viral factors required for its interaction with the arthropod were retained, for instance, those minimal components allowing for the circulative route using paracellular spaces. The study of Tetranychus urticae kitavirus (Niu et al., 2019), the closest kita-like virus infecting mites known, would likely add new elements to the mechanism underlying the movement of nege-kita-like viruses in their hosts.

If the paracellular route of cilevirus circulation in mites may be controlled in an active form, virion-membrane receptor(s) interaction has to be assumed. Accordingly, the transmissibility or not and efficiency of this process for a given pair of virus- *Brevipalpus* species must be dictated by the receptor(s) required for the virus entry into different groups of tissues by interactions with SJ. Studies on the molecular interactions between mite vectors and plant viruses are scarce (de Lillo et al., 2021). Global transcriptomic response of wheat streak mosaic virus (WSMV; genus *Tritimovirus*; family *Potyviridae*) and *Aceria tosichella* (Eriophyidae) showed the upregulation of two gene families that participate in the SJ formation (Gupta et al., 2019). Recent sequencing of the *B. yothersi* genome (Navia et al., 2019) and investigations on the function of CiLV-C-coded proteins (Leastro et al., 2018, 2020; Arena et al., 2020) may provide new insights into the putative participation of orthologues of these genes in *Brevipalpus*-cilevirus interaction.

In case the paracellular route of CiLV-C movement in *Brevipalpus* mites is confirmed, it will be the first example of a plant virus using this unconventional route of cell-to-cell movement in its arthropod vector. The unusual type of interaction with its vector, as also happens during the interaction of CiLV-C with their plant hosts, suggest that this virus, and probably other members of the family *Kitaviridae*, represent unique chimeric genetic systems that are likely reshaping features inherited from their ancestors to adapt to new ecological challenges.

## Data Availability Statement

The original contributions presented in the study are included in the article/supplementary material, further inquiries can be directed to the corresponding author/s.

## Author Contributions

AT, EK, PR-G, and JF-A: conceptualization and methodology. PR-G, AT, EK, and TS: formal analysis. JF-A and EK: funding acquisition. AT, TS, PR-G, EK, and JF-A: investigation. PR-G, EK, and JF-A: supervision. AT: writing—original draft. AT, PR-G, EK, TS, and JF-A: writing—review and editing. All authors contributed to the article and approved the submitted version.

## Conflict of Interest

The authors declare that the research was conducted in the absence of any commercial or financial relationships that could be construed as a potential conflict of interest.

## Publisher’s Note

All claims expressed in this article are solely those of the authors and do not necessarily represent those of their affiliated organizations, or those of the publisher, the editors and the reviewers. Any product that may be evaluated in this article, or claim that may be made by its manufacturer, is not guaranteed or endorsed by the publisher.

## Footnotes

1
https://blast.ncbi.nlm.nih.gov/Blast.cgi
